# The Four Epochs of Woman’s Life

**Published:** 1902-04

**Authors:** 


The Four Epochs of Woman's Life.—A study in hygiene. By Anna M. Galbraith, M. D., author of "Hygiene and Physical Culture for Women"; Fellow of the New York Academy of Medicine, etc. With an introductory note by John H. Musser, M. D., Professor of Clinical Medicine, University of Pennsylvania. 12mo volume of 200 pages. Philadelphia and London: W. B. Saunders & Company. 1901. Cloth, $1.25 net.

Some such work as Dr. Galbraith's admirable essay should be in the hands of every woman, whether married or single. Ignorance of the subjects treated of in books of this kind is responsible for most of the special diseases from which women suffer, as well as for much marital unhappiness. Every woman should have some knowledge of the menstrual function, the ethics of married life, the duties of motherhood, and the menopause. No one can find reason to object to the manner in which Dr. Galbraith discusses these all important subjects. R.

